# How Stress Alters Memory in ‘Smart’ Snails

**DOI:** 10.1371/journal.pone.0032334

**Published:** 2012-02-24

**Authors:** Sarah Dalesman, Ken Lukowiak

**Affiliations:** Hotchkiss Brain Institute, Department of Physiology and Pharmacology, University of Calgary, Calgary, Alberta, Canada; Tokai University, Japan

## Abstract

Cognitive ability varies within species, but whether this variation alters the manner in which memory formation is affected by environmental stress is unclear. The great pond snail, *Lymnaea stagnalis*, is commonly used as model species in studies of learning and memory. The majority of those studies used a single laboratory strain (i.e. the Dutch strain) originating from a wild population in the Netherlands. However, our recent work has identified natural populations that demonstrate significantly enhanced long-term memory (LTM) formation relative to the Dutch strain following operant conditioning of aerial respiratory behaviour. Here we assess how two populations with enhanced memory formation (i.e. ‘smart’ snails), one from Canada (Trans Canada 1: TC1) and one from the U.K. (Chilton Moor: CM) respond to ecologically relevant stressors. In control conditions the Dutch strain forms memory lasting 1–3 h following a single 0.5 h training session in our standard calcium pond water (80 mg/l [Ca^2+^]), whereas the TC1 and CM populations formed LTM lasting 5+ days following this training regime. Exposure to low environmental calcium pond water (20 mg/l [Ca^2+^]), which blocks LTM in the Dutch strain, reduced LTM retention to 24 h in the TC1 and CM populations. Crowding (20 snails in 100 ml) immediately prior to training blocks LTM in the Dutch strain, and also did so in TC1 and CM populations. Therefore, snails with enhanced cognitive ability respond to these ecologically relevant stressors in a similar manner to the Dutch strain, but are more robust at forming LTM in a low calcium environment. Despite the two populations (CM and TC1) originating from different continents, LTM formation was indistinguishable in both control and stressed conditions. This indicates that the underlying mechanisms controlling cognitive differences among populations may be highly conserved in *L. stagnalis*.

## Introduction

The ability of animals to learn and remember during their lifetime enables them to adapt to changes in predator threat [1], [2], food availability [3] or food quality [4], [5], as well as remembering conspecific interactions that may alter social status or mate preference [6], [7], [8], all of which will directly affect an animal's fitness. In the natural environment sub-optimal conditions can act as a stressor, considered here as changes in the environment that perturb normal physiological, psychological or behavioural function [9], [10]. This stress may significantly alter the ability of an animal to learn and form memory, dependent on the nature of the stress and timing relative to a period of learning [11], [12].

The ability to form memory can differ among populations or strains within a species. There is growing evidence that differences among populations in learning and memory is common, having been demonstrated in both vertebrates and invertebrates [13], [14], [15], [16]. Additionally, populations within a species can differ considerably in their response to a variety of environmental stimuli demonstrating local adaptation, for example in predator recognition and antipredator behaviour [17], [18], [19]. Potentially their perception of, or response to, environmental stressors that alter memory formation may also differ. In developing an understanding of the potential for a species to demonstrate behavioural plasticity through learning and memory we need to be able to assess how populations differ. These differences may occur in their ability to form memory in optimal conditions. However, populations or individuals may also be differentially affected by the environment in terms of how stress alters memory forming potential.

The great pond snail, *Lymnaea stagnalis*, has been used extensively as a model organism to study the mechanisms of learning and memory [20], [21], [22], [23]. Much of this work has been carried out using individuals that have been reared in the laboratory over many generations, derived from a population originally collected in the 1950s from canals in a polder in Utrecht province in the Netherlands (i.e. the Dutch laboratory strain). Hence, the vast majority of knowledge we now have about memory formation and factors that affect it in *L. stagnalis* is based on this single Dutch strain. However, there is growing evidence that *L. stagnalis* populations differ, both in their response to environmental stimuli [19], [24], [25], [26] and also in their ability to form long-term memory [15], [27], [28]. Cognitive ability and the response to stress is also consistent across successive generations, both in the laboratory and in the field, indicating a genetic basis to these responses [15], [29], [30].

Using the Dutch laboratory strain, the effect of stressful stimuli on long-term memory (LTM) formation differs depending on the type of stimulus used. For example, exposure to predator kairomones during training generally enhances LTM formation [31], [32]. However, other stressors such as crowding [33] and low environmental calcium [34], mediated via disparate sensory systems [35] have been shown to block LTM formation. Importantly, significant variability in LTM formation has been found among wild populations of this species. The majority of the natural populations of *L. stagnalis* tested to date exhibited identical memory forming potential to the Dutch laboratory strain following operant conditioning of aerial respiration [15], [27]. However, we have also identified populations in both Canada and the U.K. that exhibited a significantly enhanced ability to form LTM relative to the Dutch strain, and also relative to other geographically adjacent natural populations [15], [26], [27]. The memory enhancing effects of predator kairomones have been previously demonstrated across several wild populations in both Canada and the U.K. [15], [26], and appears highly conserved in *L. stagnalis*. However, it is currently unknown whether stressors that block LTM formation in the Dutch strain also have a similar effect in populations with an enhanced cognitive ability. We were therefore interested in whether *L. stagnalis* populations that demonstrate enhanced cognition differ in the effect that environmental stress has on their ability to learn and form memory relative to the standard Dutch laboratory strain.

We selected two populations that had been found to demonstrate enhanced memory formation relative to other populations in previous work, both relative to the Dutch laboratory strain and also when compared to other geographically adjacent wild populations that demonstrate an identical memory phenotype to the Dutch strain. One population, Trans Canada 1 (TC1), was sourced from a pond beside the Trans Canada Highway in Alberta, Canada [27] and one from a drainage ditch in the Chilton Moor (CM) area of the Somerset Levels, Somerset, U.K. [15]. First we assessed the duration of LTM in the absence of stressors following a single half-hour operant training session to reduce aerial respiration in hypoxia. This training regime normally results in intermediate-term memory (ITM) lasting 1 to 3 h in Dutch strain and other wild populations with a similar cognitive phenotype to the Dutch strain [15], [27], [36], [37], but results in memory lasting at least 24 h in the populations we identified as having enhanced memory retention [15], [26], [27]. Secondly, we assessed the effect of stressors that block LTM in the Dutch strain, low calcium availability [34] and crowding [33], on LTM formation in the populations with enhanced memory retention (CM and TC1). Whilst both these stressors result in the same behavioural phenotype in the Dutch strain, they are mediated via different sensory systems [35].

## Results

### Memory retention at 24 h in control conditions

**Figure 1:**
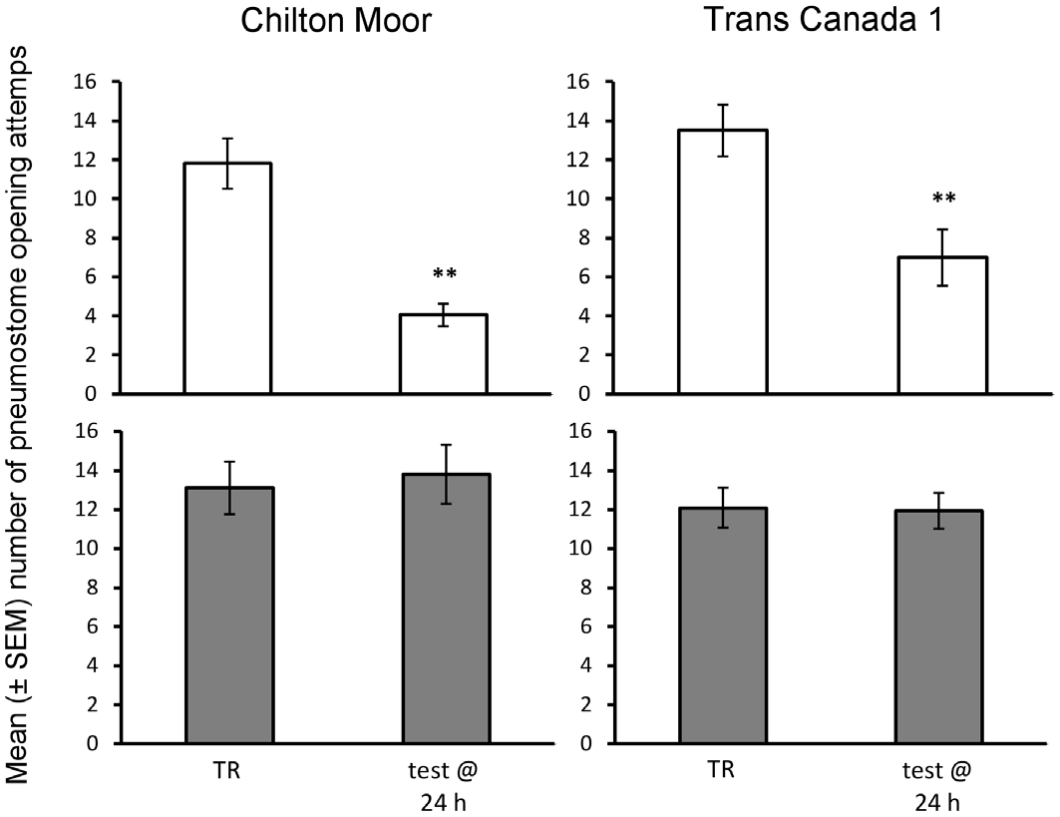
Response to operant conditioning and yoked controls at 24 h. Number of average (± SEM) pneumostome openings in 30 min during training (TR) and the test for LTM 24 h later (test @ 24 h) in the CM (Chilton Moor) and TC1 (Trans Canada 1) populations following contingent (white bars) or yoked (grey bars) training. ** = significantly different from TR (P<0.01).

There was no significant difference between the two populations (Chilton Moor, U.K.: CM vs. Trans Canada 1, Canada: TC1) within each training group, independent of the training regime. However, long-term memory (LTM) formation depended on whether the snails had received operant conditioning or the yoked control procedure (Fig. 1; rmANOVA: interaction effect between response to training and training regime: F_1,48_ = 25.91, P<0.001). Following operant conditioning (i.e. where the tactile stimulus was contingent with pneumostome opening) snails from both populations demonstrated a significant decline in breathing attempts between training (TR) and the test at 24 h (CM: t = 5.49, P<0.001, N = 16; TC1: t = 3.38, P = 0.005, N = 14). However, in yoked controls neither population showed a significant decline in pneumostome opening attempts (CM: t = −1.00, P = 0.343, N = 10; TC1: t = 0.31, P = 0.836, N = 12). There was no significant difference among training groups during TR (Student-Newman-Keuls (SNK) test: P>0.05 for all pair-wise comparisons); hence differences in the response to operant conditioning were not due to snails receiving a different number of physical stimuli.

### Duration of memory retention in control conditions

**Figure 2:**
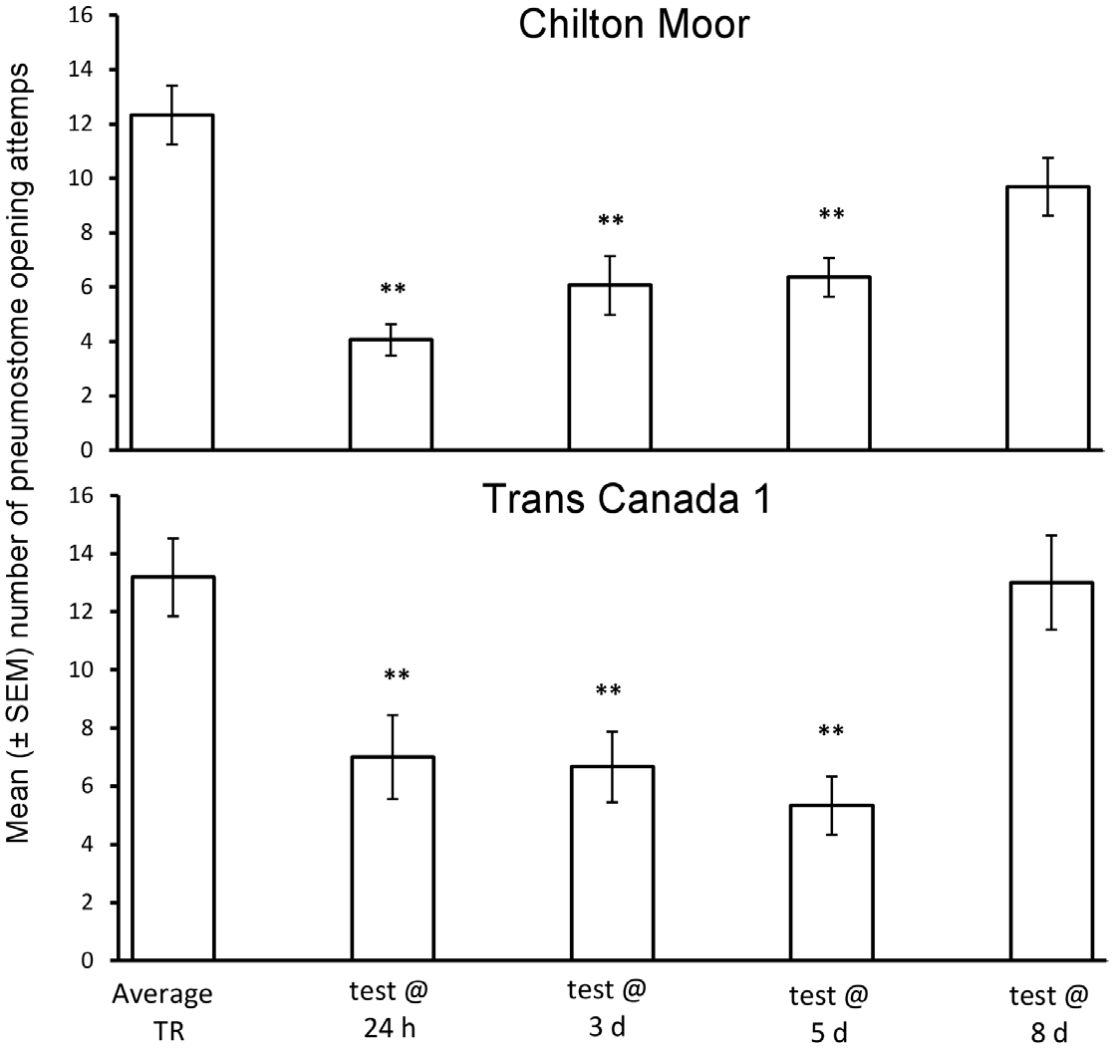
Duration of memory retention following operant conditioning. Number of average (± SEM) pneumostome openings in 30 min during training (TR: average response across four training groups is displayed) and the test for LTM (test @ 24 h, 3 d, 5 d or 8 d) in the CM (Chilton Moor) and TC1 (Trans Canada 1) populations following training in control conditions. ** = significantly different from TR (P<0.01).

There was no significant difference between populations (CM vs. TC1) or interaction effect between population of origin and time at which memory was tested on the response to training in control conditions. Whether LTM was present following training was dependant on when memory was tested (Fig. 2; rmANOVA: interaction effect between response to training and the time at which memory was tested: F_3,108_ = 5.87, P = 0.001). In all groups tested up to 5 d following training the number of pneumostome opening attempts was significantly lower during the test than during the training period for both the CM population (TR vs. test: 24 h: t = 5.49, P<0.001, N = 16; 3 d: t = 5.43, P<0.001, N = 19; 5 d: t = 5.00, P<0.001, N = 15) and the TC1 population (TR vs. test: 24 h: t = 3.38, P = 0.005, N = 14; 3 d: t = 3.76, P = 0.003, N = 12; 5 d: t = 3.66, P = 0.004, N = 12), indicating that both populations formed LTM lasting 5 d. However, at 8 d following training neither population demonstrated a significant reduction in pneumostome opening attempts relative to TR (TR vs. test at 8 d: CM: t = 1.63, P = 0.123, N = 16; TC1: t = 0.88, P = 0.399, N = 12), indicating that snails from both populations they had forgotten training by 8 d. There was no significant difference among any of the training groups in their number of attempted pneumostome openings during training (SNK: P>0.05 for all pair-wise comparisons), indicating that differences in LTM retention were not due to animals receiving a different number of physical stimuli during training.

### Low environmental calcium (20 mg/l)

**Figure 3:**
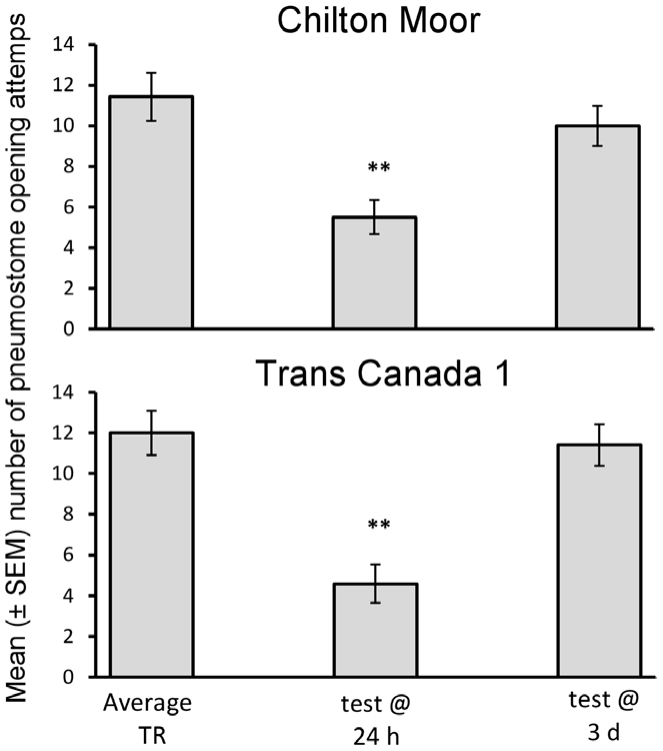
Effect of low calcium on the duration of memory retention. Number of average (± SEM) pneumostome openings in 30 min during training (TR: average response across two training groups is displayed) and the test for LTM (test @ 24 h or 3 d) in the CM (Chilton Moor) and TC1 (Trans Canada 1) populations following exposure to low environmental calcium (pale grey: 20 mg/l) for 1 week before and during training/testing. ** = significantly different from TR (P<0.01).

There was no significant effect of population of origin on LTM when snails were trained and tested in low environmental calcium. However, the time at which memory was tested significantly altered the response to training (Fig. 3: rmANOVA: interaction effect between the response to training and time at which memory was tested: F_1,42_ = 13.91, P = 0.001). When LTM was tested at 24 h both populations showed a significant decline in attempted pneumostome openings (TR vs. test at 24 h: CM: t = 3.84, P = 0.003, N = 12; TC1: t = 4.29, P = 0.002, N = 10). However, at 3 d there was no significant decline in the number of pneumostome opening attempts during the test relative to the training session (Fig. 3: TR vs. test at 3 d: CM: t = 0.69, P = 0.504, N = 12; TC1: t = 1.77, P = 0.177, N = 12). The number of initial pokes during the training session did not differ significantly between any of the treatment groups (SNK: P>0.05 for all pair-wise comparisons), therefore the difference in LTM retention was not due to differences in the initial number of physical stimuli during training. Therefore, whilst snails held in our standard calcium conditions (80 mg/l) demonstrated LTM at both 3 d and 5 d (Fig. 2), when held in low calcium (20 mg/l) memory is only apparent 24 h following training (Fig. 3).

### Crowding

**Figure 4:**
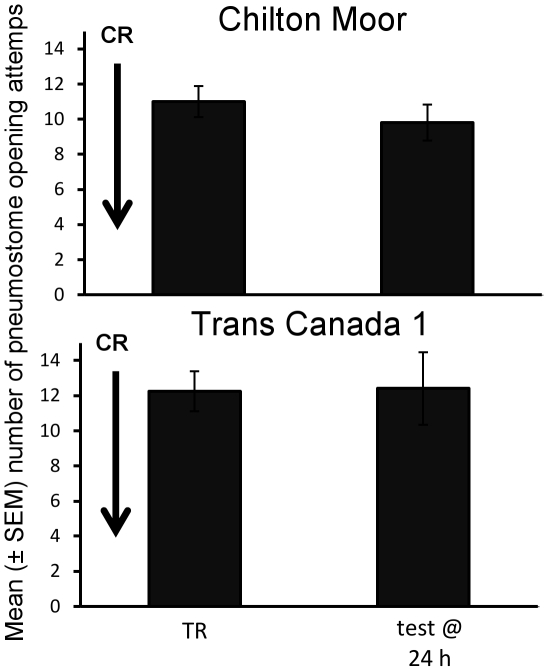
Effect of crowding on long-term memory formation. Number of average (± SEM) pneumostome openings in 30 min during training (TR) and the test for LTM 24 h later (test @ 24 h) in the CM (Chilton Moor) and TC1 (Trans Canada 1) populations following crowding (CR: 20 snails/100 ml) for 1 h immediately prior to training. There was no significant decline in pneumostome opening attempts between TR and the test at 24 h following crowding prior to TR.

Population of origin had no significant effect on the response to operant conditioning following crowding. Following crowding for 1 h immediately before TR neither population demonstrated a significant reduction in pneumostome opening attempts during the test at 24 h (Fig. 4: TR vs. test at 24 h: CM: t = 1.69, P = 0.121, N = 11; TC1: t = −0.07, P = 0.944, N = 12). As before, there was no significant difference in the number of attempted pneumostome opening between the groups during training (SNK: P>0.05 for all pair-wise tests). Therefore, whilst both populations demonstrate a significant decline in attempted pneumostome openings between TR and test at 24 h in control conditions (Fig. 2), crowding immediately prior to training prevents this decline (Fig. 4), i.e. crowding blocked LTM formation at 24 h.

## Discussion

The long-term memory (LTM) retention of both the U.K. (Chilton Moor: CM) and Canadian (Trans Canada 1: TC1) populations, when trained in the absence of stressors, is significantly enhanced relative to the ‘standard’ Dutch laboratory strain commonly used worldwide [32] and also relative to wild populations from adjacent sites in England and Canada [15], [27]. A single half-hour training session in pond water, which normally results in intermediate-term memory lasting approximately 3 h in the Dutch laboratory strain [32] and other natural populations [15], [27], produced LTM lasting 5 days in the two populations tested here. In other words, in the absence of stress memory retention lasts approximately 20 times longer in these populations with enhanced cognitive ability. Both populations, CM and TC1, demonstrated forgetting by 8 days following training. A lack of differentiation between these two populations indicates that the underlying neurophysiological phenotype enabling an enhanced ability to form and retain LTM in *L. stagnalis* populations may be the same.

Evidence for neurophysiological differentiation among populations with different cognitive ability has recently been found in Canadian populations, where differences are evident in the baseline electrophysiological activity of one of the neurons controlling aerial respiration. Right Pedal Dorsal 1(RPeD1) is a neuron in the central pattern generator (CPG) that controls aerial breathing behaviour in *L. stagnalis* [38], [39]. Changes in the activity of RPeD1 are necessary for LTM formation and retention to alter this behaviour [40]. Recent work has shown that in one of the populations used here, TC1, this neuron demonstrates some significant differences in activity relative to another geographically close population (Trans Canada 2: TC2) [27] and the Dutch strain [41]. RPeD1 appears to be ‘primed’ to form memory, showing similarities in untrained TC1 animals to that seen in individuals trained for 0.5 h that do not exhibit enhanced memory retention. Unpublished preliminary data indicates that this primed state is also found in RPeD1 in the Chilton Moor population (M.H. Braun pers. comm.). Therefore, the neurophysiological basis for differences in memory retention under control conditions following operant conditioning appears to be identical among these geographically disparate populations.

Our work thus far has found no significant differences in baseline behavioural traits (e.g. aerial breathing rate and locomotion), among populations exhibiting different LTM formation and retention [15], [26], [27]. However, the way in which these populations respond to alternate aspects of their environment may differ, co-varying with cognitive ability, and offer insights into the evolution of memory formation in *L. stagnalis*. Here, we assessed the effects of two environmental stressors that are known to block memory formation in the Dutch laboratory strain, low calcium availability [34] and crowding [33]. Crowding the snails for an hour immediately prior to training blocked the ability of both populations (CM and TC1) to form LTM. This is the same phenotypic response that we see in the Dutch strain [33], indicating that this stressor is likely to be having a similar effect on the central nervous system (CNS) in both cases. However, whilst exposure to low environmental calcium (20 mg/l) reduced the persistence of memory from 5+ days in control conditions (80 mg/l [Ca^2+^]) to less than 3 days in low calcium (20 mg/l [Ca^2+^]), unlike the Dutch laboratory strain [34], the CM and TC1 populations were still able to form LTM.

We consider that the disparity in the phenotypes produced by these different environmental stressors is likely to be due to differences among the populations in stress perception. In the Dutch laboratory strain both stressors may be perceived equally, resulting in identical memory phenotypes [35]. However, the CM and TC1 populations tested here may perceive crowding as more stressful than low calcium availability, resulting LTM being blocked in response to the former but not the latter stressor. Strain differences in stress perception differentially affecting memory have been found in other species, such as rats [42]. Perhaps the most famous example is that of maze ‘dull’ and maze ‘bright’ rats [43], which actually differ in their stress perception (i.e. anxiety in the maze) altering their ability to learn a maze rather than differing in their cognitive ability [44]. In *L. stagnalis* perception of predation threat differs among populations, which relates to the predation regime experience by each population [19]. Whilst all populations tested were able to recognise kairomones from a predatory fish and exhibit some degree of avoidance behaviour, those overlapping in distribution with the predator demonstrated a significantly enhanced response. We may also see similar local adaptation in the response to other ecologically relevant stressors, including calcium availability. We currently only have environmental calcium data at the CM site, where it was found to fluctuate between 70 mg/l and 145 mg/l over the field season between March and September. Whilst we are unable to compare this directly with either the calcium availability at the TC1 site or in the locality where the Dutch strain were sourced, average calcium availability did not differ significantly among U.K. populations in geographic proximity to the CM site that demonstrate differences in cognitive ability [15]. This indicates that average calcium availability may not be a factor influencing local variation in cognition.

Memory formation is an important factor in the ability of a species to exhibit behavioural plasticity. For example, many aquatic species rely on associative learning to recognise a predator or assess predation threat [45]. *Lymnaea stagnalis* is able to learn about predation risk [1], [46] and also enhance its ability to recognise heterospecific alarm cues through experience [47]. Additionally, it can also learn to recognise cues associated with a food source [48] or noxious stimuli [49]. Populations with enhanced cognitive ability (i.e. TC1 and CM) may be at an advantage in variable environments relative to populations not displaying enhanced cognition as behavioural plasticity is more persistent. However, the data presented here demonstrates that these populations are still vulnerable to the effects of stress reducing their ability to form LTM, and therefore potentially to demonstrate behavioural adaptation to current conditions. Together this data indicates that, in assessing the ability of *L. stagnalis* to respond to changes in their environment, we need to account for both population variability and the effects of other environmental factors, including population density and calcium availability.

Enhanced cognitive ability can carry associated costs [50], [51], and may only be selected for in natural populations where benefits derived from remembering aspects of the environment outweigh these costs. Alternatively, selection may not be acting directly on cognitive ability, but instead on other co-varying traits. In other species cognitive ability co-varies with other traits that affect fitness, for example relating to foraging behaviour. Foraging strategy and neophobia correlate with learning ability in house sparrows [52]. In *Drosophila melanogaster* an enhanced ability to form associative learning and its consolidation into LTM is associated with allelic inheritance of the *for* gene, which also alters larval foraging activity [53]. Individuals homozygote for the allele *for*(R) move more readily between food patches, show better short-term memory, but poor LTM in an associative learning task; whereas the *for*(s) allele homozygote individuals tend to be more stationary, demonstrate poorer short-term memory but better LTM. Whilst selection on these alleles may be due to their affect on memory formation, foraging activity is also selected under different environmental conditions. In high density conditions the *for*(R) allele is favoured [54], whereas frequency dependent selection acts on the *for* allele when food resources are scarce favouring individuals carrying the rarer allele [55]. Both alleles may be maintained within the population by selection on foraging behaviour, and consequently maintaining natural variation in associative memory retention. Therefore, whilst variation among populations may be apparent in one trait (i.e. ability to form LTM), other phenotypic traits that are expressed in individuals, co-varying with cognitive ability, may also be subject to selection and possibly the underlying cause of population differences in cognitive ability seen here.

Resistance to stress may be beneficial, for example it has been linked to longer life-span in both vertebrates and invertebrates [56], [57], [58], [59]. The populations tested here retain the ability to form LTM in a low calcium environment, indicating that they may be more resistant to this stressor than the Dutch laboratory strain. Natural populations can experience 3 to 10 fold fluctuations in calcium availability [60], [61], [62], and *L. stagnalis* requires environmental calcium to grow and reproduce [63], [64], [65], [66]. Therefore, acute reductions in calcium availability may be very stressful for this animal. Whilst we found no evidence that average calcium availability is a factor affecting differences in cognitive ability among natural populations in the U.K. [15], our sampling regime was not frequent enough to gain an accurate idea of how rapidly these populations experience fluctuations. A reduction in stress perception may benefit this species when calcium levels regularly fluctuate, and resistance to this stressor may be selected in rapidly fluctuating environments. If this is the case, enhanced cognitive ability may be inherited alongside calcium stress resistance. Alternatively, both stress resistance in low calcium and cognitive ability may co-vary with additional factors not yet considered, shaping population variation in these factors. Further information is required, both on the environmental variables experienced by natural populations differing in cognitive ability and also on other behavioural and physiological parameters, to elucidate selection mechanisms further.

## Materials and Methods

### Ethics statement

Ethical approval is not required for research work with *Lymnaea stagnalis*; however every effort was made to ameliorate suffering of animals, ensuring adequate food, clean oxygenated water and low density conditions. The stress treatments used here (outlined below) have no long-term effects on the animals beyond the brief exposure periods. No specific permits were required for the described field collections. The Trans Canada 1 (TC1) site is accessed via a public highway and is not situated on private or protected land. The owner of the farmland on which the Chilton Moor (CM) site is located has given permission for us to collect *L. stagnalis* at this site. The collection of *L. stagnalis* for this study did not involve endangered or protected species.

### Collection and maintenance

*Lymnaea stagnalis* adults were collected from two locations, a drainage ditch in the Chilton Moor area of the Somerset Levels, Somerset, England (CM: 51.19 N 2.88 W) and a pond adjacent to the Trans Canada highway, Alberta, Canada (TC1: 51.07 N 114.39 W). These populations were selected due to their enhanced ability to form long-term memory (LTM) relative to both the standard laboratory strain (i.e. the Dutch strain) that originates from the Netherlands, and also relative to adjacent natural populations from Alberta [27] and the Somerset Levels [15] that exhibit an identical memory phenotype to the Dutch strain. They were transported to the University of Calgary, and maintained for a minimum of 1 week prior to experiments to allow acclimation to the laboratory. Snails were maintained on a 16∶8 light∶dark schedule at 20±1°C in aerated artificial pond water (0.26 g/l Instant Ocean®, Spectrum Brands Inc. USA) in our standard calcium conditions with 80 mg/l [Ca^2+^] [34], [67] at a density of 1 snail per litre and fed romaine lettuce *ad libitum*.

### Training protocol

*Lymnaea stagnalis* are bi-modal breathers, in eumoxic conditions they breathe primarily cutaneously, absorbing oxygen from the water directly though their skin. However, in hypoxic conditions they switch to aerial breathing using a rudimentary lung opened to the air via a respiratory orifice called the pneumostome [68]. *Lymnaea stagnalis* can be trained to reduce their aerial breathing rate in hypoxic conditions by gently poking the pneumostome each time the snail attempts to open it [36], [68]. To increase snail aerial breathing rate, artificial pond water was made hypoxic (≤5% O_2_) by vigorously bubbling N_2_ through 500 ml of water in 1 litre beaker for 20 minutes before training commenced; bubbling was then continued at a reduced rate throughout training to maintain hypoxic conditions. Snails were placed into the beaker and allowed to acclimate for 10 minutes before the training session (TR). This acclimation period was then followed by a 30 minute training period using operant conditioning, such that each time a snail attempted to open its pneumostome at the water's surface the pneumostome was gently poked using a sharpened wooden stick [36], [68]. This resulted in the snail closing its pneumostome, but did not cause whole body withdrawal. To test for LTM an identical procedure to the training session was carried out 24 h or longer following TR. If the snails formed LTM the number of pneumostome opening attempts was significantly reduced during the test session (test) relative to the training session (TR).

### Memory retention at 24 h in control conditions

Firstly, we confirmed that the change in breathing attempts between TR and the test at 24 h was due to memory retention rather than a generalised response to repeated exposure to hypoxia or a physical stimulus. To confirm this we carried out yoked controls for the training procedure. Yoked control animals were paired with another snail during training, and poked in the area of their pneumostome when the snail to which they were yoked opens its pneumostome. Therefore the ‘poking’ in the yoked animal was not contingent with pneumostome opening during training. During the test phase 24 h later yoked animals were then poked contingent with pneumostome opening. If decreases in pneumostome opening were due to operant conditioning we would not expect to see a similar decline in pneumostome opening attempts in yoked animals compared to trained animals.

### Duration of memory retention

In the Dutch strain used in the laboratory [26], and also in wild populations found at adjacent sites to the TC1 and CM populations used here [15], [27] a single 0.5 h training session results in intermediate-term memory (ITM) lasting 1 to 3 h, but not LTM 24 h later. However, in populations with enhanced memory retention, a single 0.5 h training session previously resulted in LTM lasting at least 24 h [15], [27]. Whilst this previous work had demonstrated that the TC1 and CM populations have enhanced memory retention, we had not previously assessed exactly how long memory retention persists. It is necessary to have this information to be able to accurately assess the effects that different stressors potentially have on LTM in these populations. Therefore, to assess this we tested the duration of memory persistence in control conditions following a single 0.5 h training session in the TC1 and CM populations.

To assess the duration of memory retention in control conditions we trained snails from each population and the determined whether memory was present 24 h, 3 days, 5 days or 8 days following TR, using separate groups to test memory at each time period. Having confirmed using the yoked controls that a change in aerial respiratory behaviour was the result of associative learning and its subsequent consolidation into LTM, we considered that the snails still demonstrate memory for as long as their breathing rate is depressed relative to their naïve state during TR. If the number aerial breathing attempts had returned to the same level as found during TR we concluded that the snail had forgotten.

### Low environmental calcium

Low environmental calcium (20 mg/l) is considered to be adequate for the survival of wild UK *L. stagnalis* populations [64]; however we have found that maintaining snails for 1 h to 1 week in this low calcium concentration alters respiration, locomotion and memory formation in the Dutch *L. stagnalis* strain relative to those held at 80 mg/l [Ca^2+^] [34], [67]. Exposure for a week to low calcium availability blocks LTM formation in the Dutch population following two alternative training regimes, one-trial conditioning [34] or operant conditioning following two 0.5 h training sessions separated by an hour [31], [35], [69], both of which normally result in memory retained for 24 h. Here we wanted to test whether a low calcium environment also blocks LTM formation in populations that demonstrate enhanced LTM retention.

Snails were transferred in 10 l aquaria with artificial pond water containing 20 mg/l [Ca^2+^] for 1 week prior to training. Snails were then trained in low [Ca^2+^] pond water (training as above), and tested for LTM formation at either 24 h or 3 d following TR, using separate groups to test memory at each time period. The snails were maintained in low calcium conditions throughout training and testing.

### Crowding

Crowding has been found to block LTM formation in the Dutch laboratory strain when snails are crowded immediately before the training procedure [33]. Here we wanted to assess whether crowding also blocks memory formation in the TC1 and CM populations. Snails were maintained, trained and tested in our standard calcium conditions; however, immediately prior to TR snails were transferred into crowded conditions for 1 h, 20 snails (25±1 mm spire height) held in 100 ml of standard pond water in a 1 litre beaker [33]. Training was then carried out using standard training protocol (as above), and LTM tested at 24 h.

### Data analysis

Data were analysed using repeated measures ANOVA (rmANOVA) in SPSS 17.0 (SPSS Inc., Chicago, IL, USA). Homogeneity of variance was confirmed using Mauchly's test for sphericity prior to analysis. Where overall significance was found, post-hoc paired t-tests were used to assess within-subject pair-wise differences (TR vs. test) and Student-Newman-Keuls (SNK) tests were used to assess between-subject pair-wise differences. No individual snail was trained and tested more than once throughout. For Figures 2 and 3, where different individuals were used to assess memory retention at different time points, the TR presented is the mean number of pneumostome opening attempts for all groups combined, though the individual TR for each group was used for statistical analysis.

To assess whether changes in breathing attempts were due to memory retention or a general response to repeated exposure to hypoxia or physical stimulus we used yoked controls. The response of individuals to training was used as the within-subject factor (TR vs. test at 24 h), training protocol (trained vs. yoked) and population of origin (CM vs. TC1) were used as the between-subject factors.

The duration of LTM retention in control conditions was assessed by comparing the response to training as the within-subject factor (TR vs. test), with the duration between training and testing (24 h vs. 3 d vs. 5 d vs. 8 d) and the population of origin (CM vs. TC1) as between-subject factors.

The effect of low calcium exposure on memory retention was assessed by comparing memory at 24 h and 3 d following exposure to low calcium (20 mg/l) for one week prior to and during training and testing. The response to training (i.e. LTM formation) was used the within-subject factor (TR vs. test), the between-subject factors used were the duration between training and testing (tested at 24 h or 3 d) and the population of origin (CM vs. TC1).

To analyse the effect of crowding individuals immediately prior to training, LTM formation 24 h following TR was assessed in individuals that had been held in crowded conditions for 1 h before training. The within-subject factor was the response to training (TR vs. test) and population of origin (CM vs. TC1) was used as the between-subject factor.
